# Translational lessons from the balanced immune system in bats

**DOI:** 10.1242/dmm.050763

**Published:** 2025-02-19

**Authors:** Wei Lun Ng, Lin-Fa Wang

**Affiliations:** Programme in Emerging Infectious Diseases, Duke-NUS Medical School, Singapore 169857, Singapore

**Keywords:** Bat biology, Bat immunology, Emerging infectious diseases

## Abstract

Bats are a natural reservoir for a wide variety of notorious viruses that are deadly to humans and other mammals but cause no or minimal clinical damage in bats. The co-evolution of bats and viruses for more than sixty million years has established unique and balanced immune defenses within bats against a number of viruses. With the COVID-19 pandemic, bats have gained greater attention as a likely reservoir of the SARS-CoV-2 ancestor virus. The coupling of omics technology and bat research opens an exciting new field to understand and translate discoveries from bats to humans, in the context of infectious disease and beyond. Here, we focus on the mechanism of immunity balance in bats, the application of omics and how this might lead to improvement of human health.

## Teaser

**Summary:** The balanced immune response of bats after viral infection together with the application of high-throughput technologies enables discoveries with high translational value that can improve human health and prepare for future pandemics.

## Introduction

Bats are a unique group of mammals with the ability of self-powered flight, vocal learning, healthy longevity with low occurrence of cancer, and unique immunity. Bats are a diverse group, accounting for more than 20% of mammalian species and comprising >1400 species that are distributed across every continent except Antarctica (Teeling et al., 2018). Despite playing an important role in insect control, pollinating plants and reseeding deforested lands, bats are often negatively perceived as a threat to human health because of their reputation as major transmitters of zoonotic viruses. Although it was under debate for many years, recent analysis has shown that bats are, indeed, a unique virus reservoir (Olival et al., 2017; Wang and Anderson, 2019). Bats host a higher proportion of zoonotic viruses than other mammals (Olival et al., 2017; Luis et al., 2013; Brook and Dobson, 2015), although there have been contradicting findings (Mollentze and Streicker, 2020). Despite being a host for a variety of viruses−including Hendra, Nipah, Marburg, SARS-CoV-1, and the suspected origin for MERS-CoV, Ebola, and SARS-CoV-2 viruses (Wang et al., 2020) – and these pathogens causing high lethality in humans and domestic animals, they cause only minimal or no clinical diseases in bats. There are many factors making bats an excellent reservoir of viruses. These include ecology diversification, widespread species and social interaction. However, more recently, it has become evident that the unique bat immunity may be a more important contributor (Irving et al., 2021).

Evolution of the balanced immune system of bats can be attributed to several factors, such as adaptation to flight and co-existence with viruses. Understanding how bats control virus-mediated pathogenesis with balanced immune responses helps identify therapeutic targets to treat infection with these viruses in other mammals, including humans. This Perspective highlights some key features of the balanced bat immune system and discuss innovative approaches to expand translational research in bats.

## Genomic and transcriptomic features of the bat immune system

Mammalian cells have evolved innate immune-sensing ability through highly conserved pattern recognition receptors (PRRs) that recognize pathogen-associated molecular patterns (PAMPs) derived from bacteria, viruses and parasites (Fitzgerald and Kagan, 2020). Infected cells then initiate a signaling cascade to induce the expression of thousands of antiviral and pro-inflammatory cytokines via the inflammasome (Box 1), interferon (IFN) (Box 1) or the nuclear factor kappa B (NF-κB) signaling pathways (Box 1) (Kawai and Akira, 2006). Innate immunity in humans and rodents has been investigated extensively, and recent discoveries have uncovered similar pathways in bats. As part of the pioneering effort in detecting homologs of innate immune system components in bats, analysis using several transcriptomic datasets have identified a wealth of immune-related genes in bats. This analysis revealed that 3.5% of transcribed genes in the bat species *Pteropus alecto* (Papenfuss et al., 2012) and 2.75% in that of *Rousettus aegyptiacus* (Lee et al., 2015) have immune-related functions − compared to 7% of the human genome encoding immunoproteins (Kelley et al., 2005). Although the annotation quality of bat genomes needs yet to be improved, it is possible that there are more bat-specific non-annotated transcripts or a wider range of immune genes to be explored in the bat genome, or that bats have a smaller pool of immune-related transcripts compared to human. Thus, further in-depth research and validation is required.

**Box 1. Glossary**

**APOBEC3 genes**: Members of the apolipoprotein B mRNA-editing enzyme catalytic subunit catalytic polypeptide (APOBEC) superfamily of genes that encode antiviral DNA cytidine deaminases that suppress diverse viruses.

**ASC (apoptosis-associated speck-like protein containing a CARD; also known as PYCARD):** Adaptor protein in the inflammasome pathway enabling its activation.

**ASC2 (pyrin domain-containing protein 1; also known as PYDC1):** ASC-interacting protein that inhibits inflammasome activation.

**Bat-mouse bone marrow chimera:** Animal model used to investigate the immune system of the bat, for which bat bone marrow cells are transplanted into an immunodeficient mouse strain.

**Complement genes:** Set of >45 genes making up the complement system, consisting of distinct plasma proteins that help to eliminate microbes and damaged cells through local inflammatory responses.

**IDO1 (indoleamine 2,3-dioxygenase 1):** Enzyme that catalyzes the degradation of tryptophan to N-formyl-kynurenine and has an antimicrobial role, and a role in anti-tumor immunity and immunoregulation.

**Immunosenescence:** Deterioration of immune cell function associated with aging.

**Inflammasome:** Inflammasomes are cytosolic multiprotein complexes that are assembled upon detection of infection or stress, leading to activation of inflammatory responses.

**Interferons (IFNs):** IFNs are a group of signaling molecules, such as cytokines, that are released in response to viral infections. IFNs are classified into three types − type I (IFNs α, β, ε, κ and ω), type II (IFNγ) and type III (IFNλ) − based on the receptors they bind and signal through.

**Killer cell lectin-like receptors and immunoglobulin-like receptors (KLRs and KIRs, respectively):** Two distinct structural families of natural killer (NK) cell receptors, both comprising proteins that regulate cytotoxic NK cell activity.

**NF-κB:** The NF-κb family of transcription factor protein complexes is crucial for the induction of a large number of pro-inflammatory genes and a central mediator of priming signals for NLRP3 inflammasome activation.

**Poly I:C (polyinosinic:polycytidylic acid):** Synthetic analog of double-stranded RNA that is used to simulate viral infections.

**PYHIN (pyrin and HIN domain) gene family:** Family of genes encoding intracellular DNA immune sensors − such as AIM2, IFI16, MNDA and POP3 – that activate inflammasome or/and interferon responses.

**RNASEL (ribonuclease L):** Interferon-induced ribonuclease that mediates antiviral effects by direct cleaving of RNA.

To fully capture the molecular basis of how bats adapt to viruses, six quality reference genomes were coupled with a genome-wide screen to reveal a loss of immunity-related gene regulators (e.g. NF-κB) and expansion of APOBEC3 genes (Box 1) (Jebb et al., 2020), both of which might contribute to the unique immunity of bats. Additionally, one of the most-crucial viral defense lines, the IFN system, varies between bat species (Banerjee et al., 2020). Comparative analysis of two different bat species−*Myotis davidii* and *Pteropus alecto* – has demonstrated positive selection of a high proportion of DNA damage checkpoint and DNA repair genes, loss of the entire PYHIN gene family (Box 1), and the absence of killer cell lectin-like receptors (KLRs) and killer cell immunoglobulin-like receptors (KIRs) (Box 1) (Zhang et al., 2013). Moreover, recent single-cell RNA sequencing (scRNA-seq) data obtained from the Egyptian fruit bat showed unique expression of complement genes (Box 1) within lung, gut and blood (Levinger et al., 2023). Cross-species scRNA-seq was also used to delineate species-specific host responses to pathogens, as inoculating peripheral blood mononuclear cells (PBMCs) with herpes simplex virus (HSV-1), Sendai Virus (SeV) or lipopolysaccharides (LPS), has identified a new subset of monocytes in *Rhinolophus aegyptiacus* (Aso et al., 2022).

## *In vivo* infection studies in bats

Bats host diverse zoonotic viruses. Experimental infection of bats with Ebola (Paweska et al., 2016), Marburg (Amman et al., 2015; Schuh et al., 2017; Guito et al., 2021), MERS (Munster et al., 2016), Nipah (Middleton et al., 2007) and Hendra virus (Halpin et al., 2011; Woon et al., 2020), showed limited viraemia and, remarkably, no major disease symptoms, immunopathology or death. This scenario contrasts with the severe morbidity and mortality experienced by humans following infection with these viruses. Transmission studies speculated that fruits bats possess the characteristics of a reservoir host. Importantly, captive bat breeding colonies allow the study of *in vivo* infection in a more controlled environment, with known pathogen exposure history, compared to study of bats caught in the wild. Recent scRNA-seq characterization of the immune responses to infection with *Pteropine orthoreovirus* of captive colony bats *Eonycteris spelaea* showed a broad NK and T cells activation, and high basal levels of IDO1 (Box 1) limited to neutrophils (Gamage et al., 2022). Interestingly, cross-species comparisons of published data from human, mouse and pig lung tissues did not show significant IDO1 expression in neutrophils (Gamage et al., 2022). This suggests that bats harbor a distinct mechanism to limit inflammation by expressing IDO1 in neutrophils. Moreover, bat immune responses to viruses differ from humans, particularly regarding interferon and antiviral activity. For example, bats constitutively express *Ifna* (Zhou et al., 2016) and several antiviral genes. Overall, expression profiles of *Ifna* and *Ifnb* are unique in bats compared to those in human or mice, indicating bat-specific changes to control viruses (Shaw et al., 2017; Glennon et al., 2015; Holzer et al., 2019). Furthermore, RNASEL (Box 1) is inducible upon viral infection in bats but not in other mammals (De La Cruz-Rivera et al., 2018).

Another key mechanism of the bat immune system is the dampening or prevention of immune over-responses to complement antiviral responses. For instance, loss of the PYHIN gene family (Ahn et al., 2016), dampened activation of NLRP3 (Ahn et al., 2019), reduced caspase-1 activity (Goh et al., 2020) and inhibitory function of ASC2 (also known as PYDC1) (Box 1) in the bat inflammasome pathway (Ahn et al., 2023) all contribute to dampening inflammatory responses in bats. Less robust induction of TNF (Banerjee et al., 2017) and STING mutants in bats further support this reduced inflammation during infection (Xie et al., 2018). In short, the uniqueness of their balanced immune system renders bats to better co-exist with various viruses, as summarized in Table 1.

**Table 1.** Features of the balanced bat immune system

Gene/gene family	Functional context	Disease context	Bat species studied	Reference
***Aim2*, *Ifi16***	Genomic loss of these PYHIN gene family members in ten bat species	n/a	Ten bat species^#^	Ahn et al. (2016)
***Asc2***	Inhibits ASC in the inflammasome pathway	Immunostimulation in response to poly dA:dT, Talabostat, members of the flagellin protein family, the antibiotic nigericin, MSU crystals. Infection with IAV, ZIKV, PRV3M or SARS-CoV-2 immune complex	*P. alecto*	Ahn et al. (2023)
***Casp1***	Reduced activity and complementary regulation with IL1B	n/a	*P. alecto*	Goh et al. (2020)
***Ifnar2***	First CRISPR knockout attempt in bat cell lines. Indispensable in bat IFNA signaling	Novel IFNAR2-dependent IRGs related to cancer. Infection with Influenza virus H1N1	*P. alecto*	Zhang et al. (2017)
***Ifna* gene family**	Three IFNA genes are constitutively expressed, IFNA3 is functional. Shortened type I IFN locus in the bat genome, comprising only ten IFN loci.	Infection with bat reovirus PRV1NB	*P. alecto*	Zhou et al. (2016)
	Constitutive expression not observed	n/a	*R. aegyptiacus*	Pavlovich et al. (2018)
***Ifnb1***
	Increased induction of IFNB1 but reduced induction of TNF	Mimicking of viral infection with poly I:C	*E. fuscus*	Banerjee et al. (2019)
	Increased induction of *IFNB1* and TNF	Mimicking of viral infection with poly I:C or LPS	*M. myotis*	Kacprzyk et al. (2017)
	Induced upon poly I:C but not HeV, NiV-M or NiV-B	Mimicking of viral infection with poly I:C, infection with HeV, NiV-M or NiV-B	*P. alecto*	Virtue et al. (2011)
	Induction levels of *IFNB1* in bats are higher compared to those in in mice	Mimicking of viral infection with poly I:C; infection with VSV	*R. affinis*, *R sinicus*	Li et al. (2015)
***Ifnk***	Limits replication of lyssavirus strains	Infection with lyssavirus	*E. serotinus*	He et al. (2014)
***Ifnl***	Encodes three type III IFNs	n/a	*P. vampyrus*	Zhou et al. (2011)
	Upon infection, expression of *IFNL1* and *IFNL2* but not type I IFN in *P. alecto.*	Infection with Tioman virus	*P. alecto*	Zhou et al. (2011)
***Ifnw***	Induced upon infection	Infection with Sendai virus	*R. aegyptiacus*	Pavlovich et al. (2018)
	Limits replication of lyssavirus strains	Infection with lyssavirus	*E. serotinus*	He et al. (2014)
***Il10***	Pro-inflammatory response sustained by high levels of IL10	LPS, Poly I:C	*M. myotis*	Kacprzyk et al. (2017)
***Isg54*, *Isg56, Il28*, *Il29*,**	Induced upon infection with poly I:C but not HeV, NiV-M or NiV-B	Mimicking of viral infection with poly I:C, infection with HeV, NiV-M or NiV-B	*P. alecto*	Virtue et al. (2011)
***Irf3***	Antiviral signaling upon infection with poly I:C and MERS-CoV	Infection with MERS-CoV and poly I:C to mimic viral	*E. fuscus*	Banerjee et al. (2019)
***Irf7***	More widespread mRNA distribution of *IRF7* compared to that in human and mice. Knockdown of *IRF7* reduces levels of IFNB following infection. Increased viral titer following infection.	Infection with SeV Infection with PulV	*P. alecto*	Zhou et al. (2014)
***Lgp2*, *Mda5*, *Rig-I*,**	Tissue-specific expression levels and primary structure are comparable to those in human cells	Infection with poly I:C to mimic viral infection	*P. alecto*	Cowled et al. (2012)
***Mavs***	Functionally conserved between human and mice	Expression of hepatovirus 3ABC proteases leads to cleavage of human MAVS	*R. sinicus*, *E. helvum*	Feng et al. (2019)
***Mx1*, *Oas1*, *Pkr***	Of these three IFN-induced genes studied, highest induction was of *Oas1.* Bat *Oas1* contains two IFN-sensitive response elements (ISREs) compared to one in human *OAS1.*	Infection with bat reovirus PRV1NB	*P. alecto*	Zhou et al. (2013)
***Nlrp3***	Reduced activation in bat immune cells	Priming with LPS+ATP or nigericin. Infection with IAV, bat reovirus PRV3M or MERS-CoV	*P. alecto*	Ahn et al. (2019)
***Sting***	Mutation at serine residue 358 dampens interferon activation	Immunostimulation in response to cGAMP	*P. alecto*	Xie et al. (2018)
***Tlr3***	Poly I:C triggers signaling via TLR3 to activate transcription of *Ifnb* in *E*. *fuscus* kidney cells	Mimicking of viral infection with poly I:C	*E. fuscus*	Banerjee et al. (2017)
***Tlr8***	Owing to functional constraints, 63% of bat TLR8 undergoes purifying selection; sequence varies between bat species	n/a	21 bat species*	Schad et al. (2016)
***Tlr9***	Reduced potency of TLR9 in bat compared with that in human cells.	Immunostimulation in response to CpG ODN	*E. fuscus*	Banerjee et al. (2017)
	Eight bats from three families harbor multiple positively selected mutations within *TLR3*, *TLR8* and *TLR9*.	n/a	* *	Escalera-Zamudio et al. (2015)
***Tnf***	Repressor (c-REL)-binding motif within the *TNF* promoter	Mimicking of viral infection with poly I:C	*E. fuscus*, *M. davidii* (predicted), *M. natalensis* (predicted)	Banerjee et al. (2017)
***Trim40***	Induced upon infection. Knockdown of TRIM40 correlates with reduced viral titers	Infection with Nipah virus infection	*P. vampyrus*	van Tol et al. (2024)

cGAMP, cyclic GMP-AMP CpG ODNs (immunostimulant); CpG oligodeoxynucleotides (promoting antigen-specific immune responses); hepatovirus 3ABC proteases, inhibitors of antiviral signaling; HeV, Hendra virus; IRGs, interferon regulated genes; IAV, influenza A virus; IFNA, interferon alpha proteins; IFNB, interferon beta proteins; n/a, not applicable; Poly dA:dT, poly(deoxyadenylic-deoxythymidylic) acid sodium salt (immunostimulant); poly I:C, polyinosinic:polycytidylic acid (immunostimulant); PRV1NB, Pteropine orthoreovirus NB; PRV3M, Pteropine orthoreovirus 3M (also known as Melaka virus); PulV, Pulau virus; SeV, Sendai virus; ZIKV, Zika virus.

*N. *noctula*, *N*. *leisleri*, *E*. *fuscus, P*. *nathusii, V*. *murinus*, *M*. *myotis*, *M. davidii*, *M. brandtii*, *M. lucifugus*, *V. bidens*, *V. pusilla*, *C. perspicillata*, *P. parnelli*, *N. albiventris*, *N. leporinus*, *S. bilineata*, *R. euryale*, *R. ferrumequinuum*, *R. hipposideros*, *E. helvum*, *P. alecto*.

^#^*P. alecto*, *P. vampyrus*, *E. helvum*, *R. ferrumequinum*, *M. lyra*, *M. davidii*, *M. brandtii*, *M. lucifungus*, *E. fuscus*, *P. parnelli*.

## Future perspective: Translating lessons from the balanced immune system in bats to human health

One of the first promising approaches to further explore the bat immune system is studying the landscape and dynamics of immune cells across ages. Immune tolerance restricts host-mediated pathology in young infants (Kollmann et al., 2017); thus, studying age-related differences in bat immunity could improve our understanding of viral fitness, maintenance and spill over. Recent reports unveiled a substantial enrichment of CD79A^+^ (i.e. CD79A-positive) B cells and CD11B^+^ T cells in juvenile animals compared to those in older bats, while neutrophils, CD206^+^ myeloid cells and CD3^+^ T cells make up the majority of cell types as bats enter adulthood (Friedrichs et al., 2022). Furthermore, it is worth noting that a cumulative risk of Hendra virus persistence and maintenance with age was found in *P. scapulatus* after waning of maternal immunity, reflecting a distinct host-pathogen relationship (Plowright et al., 2011).

**Fig. 1.**
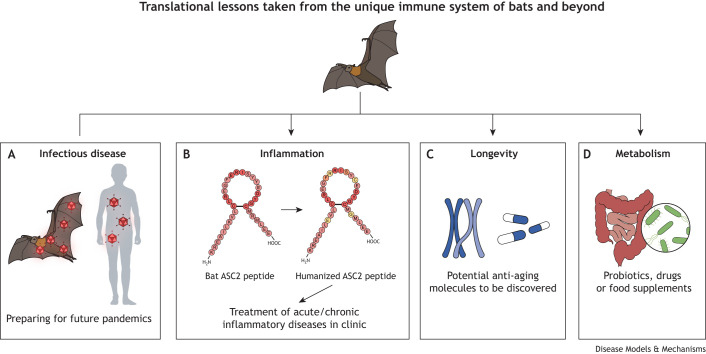
**Translational value of bat research to humans.** (A-D) Investigating emerging infectious disease (A), inflammation (B), longevity (C), metabolism (D).

An important question is whether immune cell aging contributes to the immune tolerance in bats. During the COVID-19 pandemic, elderly people were more likely to suffer from severe cytokine storm and had a higher mortality rate. In general, increased infection susceptibility, poor vaccine response and age-related diseases are caused by immunosenescence (Box 1) (reviewed by Liu et al., 2023). Additionally, aged immune cells also promote solid organ aging, and these aged organs secrete senescence-associated secretory phenotype (SASP) factors and cause chronic inflammation. Importantly, removal of senescent cells ameliorates SARS-CoV-2 symptoms (Delval et al., 2023; Camell et al., 2021), improves organ transplant outcomes (Iske et al., 2020) and delays age-related disorders (Baker et al., 2011). Since bats exhibit a multi-layered inflammation dampening strategy and reduced senescence (Ricklefs, 2010; Fleischer et al., 2017), studying immune cell aging in bats is a new and fertile realm to, potentially, develop alternative treatment strategies for viral infections and to better support an ageing population (Fig. 1A).

Bat immunology, albeit at its infancy, has already offered great insights into treating human inflammation. For example, bat ASC2 interacts with ASC (also known as PYCARD) (Box 1) to dampen inflammasome responses (Fig. 1B), and substitution of four key residues in human ASC2 (i.e. PYDC1) allows it to significantly inhibit inflammation *in vitro* and *in vivo* (Ahn et al., 2023). Following on from this study, our group is further investigating the mechanisms of these processes by applying these results in human tissues via the development of different drug candidates to dampen inflammation. Such therapeutics can be harnessed for many inflammatory diseases, such as infectious or age-related diseases, including cardiovascular and neurodegenerative diseases.

With the recent surge of investment in longevity research, aging – not just in the context of infectious disease responses−has garnered huge interest (Fig. 1C). A dampened immune system might be a by-product of the healthy lifespan of bats, as they are unparalleled in their relationship between body mass and longevity − with up to ten times longer lifespan than similar sized land mammals. The oldest bat (belonging to the species *Myotis brandtii*) is >41 years old, weighing only 7 g, and is suspected to comprise low tumorigenesis (Podlutsky et al., 2005; Gorbunova et al., 2020). Longitudinal studies using captive breeding colonies of known age will be useful to compare aging mechanisms − as, for instance, epigenetic clock determination − between bats and human or rodents (Wilkinson et al., 2021). Long-lived *Myotis* bats seem to harbor the alternative lengthening of telomeres (ALT) mechanism, as they can maintain telomere length without telomerase expression (Foley et al., 2018). Analysis suggested that DNA repair genes were involved in telomere maintenance and contribute to the extreme longevity, which represent therapeutic targets for cancers and aging process (Foley et al., 2018).

Balanced antiviral and inflammation control in bats provides valuable translational lessons to ameliorate human health in acute and/or chronic inflammation, age-related and metabolic diseases

Metabolic disease is another attractive area to study in bats (Fig. 1D). It is well known that certain bats feed on high levels of sugars while avoiding metabolic diseases. The metabolic adaptation to process large quantities of sugars enables fruit bats to exploit energy-rich diets to fuel their rigorous flying activities. By using fruit and nectar feeding bats, several research groups have analyzed sequencing data (Fang et al., 2014; Potter et al., 2021; Shen et al., 2012) and interrogated single cells in bats kidneys and pancreas to uncover mechanisms with a therapeutic potential for metabolic diseases, such as diabetes (Gordon et al., 2024). Given the different feeding behaviors among bat species as well as the gut microbiome and its association with host health, further analysis of metabolism in bats could enable probiotic developments beneficial for human health. Comparative analysis using customized high-fat diets on bats and rodents provide a way to assess how the microbiome of bats changes and how feeding strategies allow them to stay healthy while being a zoonotic reservoir. Balanced antiviral and inflammation control in bats provides valuable translational lessons to ameliorate human health in acute and/or chronic inflammation, age-related and metabolic diseases.

## Future approach: Expanding the toolbox for bat research

**Fig. 2.**
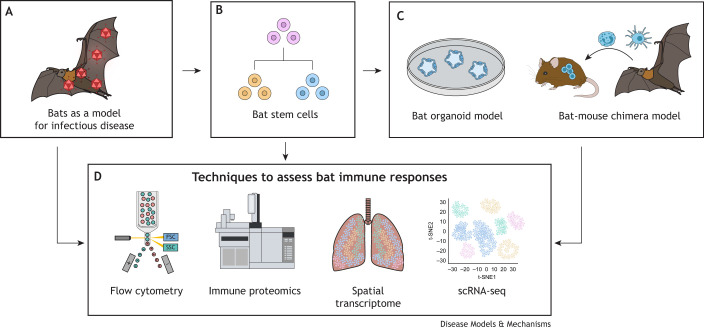
**Future approaches, tools and challenges in bat immunology research.** (A) Bats serve as a valuable model organism for infectious disease by studying their unique immune system and its regulation. (B) The advancement of bat stem cells for future disease model investigation. (C) Potential development of organ-specific organoids and a bat-mouse chimera model to study the immune system of bats in mice. (D) Techniques to assess the bat immune system, such as organ-specific high-throughput sequencing methods to cluster immune cell subpopulations in bats.

Model organisms like non-human primates and rodents are well established for studying disease pathogenesis. However, non-model organism, such as bats, can be excellent systems for discovering disease resistance mechanisms (Fig. 2A). Strikingly, bats are more closely related to humans than mice in terms of their repertoire of immune-related genes (Gamage et al., 2020). This genetic similarity encourages the translation of lessons learned from bats to clinical treatments in humans.

Several bat-breeding colonies have been established worldwide, including *E. spelaea*, *A. jamaicensis*, *R. aegyptiacus* and *E. helvum* colonies, which are valuable in deriving fresh samples to study different research questions ranging from inflammation, metabolism, longevity and cancer both *in vivo* and *in vitro*. Bat stem cells can also help not only solve the issue of bat breeding colonies for other species that are challenging to establish, but also can solve the limited lifespan of bat primary cell lines (Déjosez et al., 2023) (Fig. 2B). Importantly, pluripotent stem cells can differentiate into any cell type and are amenable for genetic modification, such as CRISPR editing. The possibilities opening by using bat stem cells are endless, not only to serve as a model to study bat biology in general, but also to elucidate virus diversity and the molecular adaptations that render bats to asymptomatically host these viruses. Stem cells will also be highly valuable to establish ‘batized’ mice, i.e. bat-mouse chimera systems that are more-defined compared with the first generation of bat-mouse bone marrow chimeras (Box 1), created by using bat bone marrow (Yong et al., 2018). Also, organoids of different human and bat tissues can be developed to study viral infection to shed light on the tissue tropism, and to understand what confers bat immune tolerance to viruses (Fig. 2C).

Despite the development of these innovative models, a major issue in bat immunology research is the paucity of species-specific tools. ScRNA-seq has provided a high-resolution platform to partially overcome the scarcity of bat-specific immunology tools, which can be harnessed alongside the development of bat-specific antibody panels for flow cytometry and western blotting. Although scRNA-seq provides marker-free decomposition of cell types and subtypes from tissues or blood for cell types and subtype identification, its cost and capture efficiency remains a limitation. Nevertheless, as spatial single-cell transcriptomics is getting more common, multiple bat organs and blood can be sequenced to better understand environmental interactions and distinct immune cell pools in bats that harbor specific viruses (Fig. 2D).

These approaches create a toolbox of current bat research that can be deployed to better prepare for the next pandemic

Proteomics is another challenging issue faced by the bat community due to the differing genome, open reading frame (ORF) and peptide annotation amongst bat species. Proteomics is promising in cataloguing the factors involved in bat responses upon infection, age-related cytokine production and metabolic assays, since the blood volume required is minimal for these experiments, enabling longitudinal studies without the need for lethal sampling. Targeted mass spectrometry of the plasma proteome has been attempted in several bat species (Hecht et al., 2015; Ahn et al., 2019), yet the profiling of immune cells requires further refinement that could be achieved by adapting rapid proteomic techniques used for human plasma analysis (Geyer et al., 2016). Specifically, serum proteomics have been attempted in vampire bats (*Desmodus rotundus*) by using data-independent acquisition (DIA), which provide a framework that is applicable across bat species and future pathogen surveillance (Neely et al., 2021; Vicente-Santos et al., 2023). Applying a proteomic approach across different organs in different bat species could improve our knowledge of which bat species can tolerate viruses and how they confer this tolerance (Fig. 2D). Omics technology could also be harnessed in bat-mouse bone marrow chimera systems (Yong et al., 2018), the other powerful animal model to study the unique immunological functions in bats.

These approaches create a toolbox of current bat research that can be deployed to better prepare for the next pandemic. Consequently, unlocking immune mechanisms that are employed by bats to dampen disease severity would be highly translatable to humans.

At this stage, the scientific community − including bat researchers and industry partners − should collaborate more extensively regarding funding, technology exchange and cutting-edge tools used in bat research, to prepare for future pandemics

## Concluding remarks

Flight adaptation and unique immune responses in bats allowing them to co-exist with viruses has provided lessons we could translate into clinical settings. The bat immunology field is still at its infancy yet burgeoning post pandemic. At this stage, the scientific community − including bat researchers and industry partners − should collaborate more extensively regarding funding, technology exchange and cutting-edge tools used in bat research, to prepare for future pandemics. As the catalogue of genomic and phenotypic characterization of pan-bat species are expanding, we should mechanistically study the specific features of various bats species in regard to tolerating viral infection and sustaining healthy aging, and in controlling excessive inflammation that leads to age-related and metabolic diseases. To achieve progress in translating findings from bats to humans, sustainable funding mechanisms and open-source sharing platforms for bat researchers are needed.
